# Supplementary material for: Cerebrolysin Prevents Brain Injury in a Mouse Model of Liver Damage
Source: Brain Sci. 2021 Dec 9;11(12):1622. doi: 10.3390/brainsci11121622 (PMC8699728; doi:10.3390/brainsci11121622)
Supplement: Supplementary file 1 [file brainsci-11-01622-s001.zip › brainsci-1471876 supplement.pdf]

brain sciences

MDPI

Supplementary Materials

Cerebrolysin prevents brain injury in a mouse model of liver damage

Shandiz Morega [1], Bogdan Cătălin [2]*, Cristiana Eugenia Simionescu [3]*, Konstantinos Sapalidis [4] and Ion Rogoveanu [5],

[1] U.M.F. Doctoral School Craiova, University of Medicine and Pharmacy of Craiova, Romania, 200349 Craiova; morega.shandiz@yahoo.com; morega.shandiz@yahoo.com
[2] Experimental Research Centre for Normal and Pathological Aging, University of Medicine and Pharmacy of Craiova, Romania, 200349 Craiova, Romania; bogdan.catalin@umfcv.ro
[3] Department of Pathology, University of Medicine and Pharmacy of Craiova, Romania, 200349 Craiova, Romania; csimionescu2004@yahoo.com
[4] 3rd Department of Surgery, AHEPA University Hospital, Medical School, Aristotle University of Thessaloniki, Thessaloniki, Greece; sapalidis@med.auth.gr
[5] Gastroenterology Department, University of Medicine and Pharmacy of Craiova, 200349 Craiova, Romania; ionirogoveanu@gmail.com
* Correspondence: bogdan.catalin@umfcv.ro and csimionescu2004@yahoo.com

Abstract. Liver damage can lead to secondary organ damage by toxic substances and catabolic products accumulation which can increase the permeability of blood-brain barrier, leading to cognitive impairment. The only real treatment for end stage liver failure is grafting. With some, but not all, neurological symptoms subsiding after transplantation, the presence of brain damage can impair both the short and long-term outcome. We tested if Cerebrolysin can prevent brain injury in an experimental model of non-viral liver damage in mice. Behavior, abdominal ultrasound evaluation and immunohistochemistry were used to evaluate the animals. No ultrasound or behavior differences were found between the control and treated animals, with both groups displaying more anxiety and no short-term memory benefit compared to sham mice. Cerebrolysin treatment was able to maintain a normal level of cortical $NeuN^+$ cells and induced an increase in the area occupied by $BrdU^+$ cells. Surprisingly, no difference was observed when investigating $Iba1^+$ cells. With neurological complications of end-stage liver disease impacting the rehabilitation of patients receiving liver grafts, a neuroprotective treatment of patients on the waiting lists might improve their rehabilitation outcome by ensuring a minimal cerebral damage.

Keywords: liver transplantation, neurological complications, neuroprotection

Citation: Morega, S.; Cătălin, B.; Simionescu, C.E.; Sapalidis, K.; Rogoveanu, I. Cerebrolysin prevents brain injury in a mouse model of liver damage. *Brain Sci.* **2021**, *11*, 1622. https://doi.org/10.3390/brainsci11121622

Academic Editor(s): Giovanni Grasso

Received: 04 November 2021
Accepted: 06 November 2021
Published: 09 December 2021

Publisher's Note: MDPI stays neutral with regard to jurisdictional claims in published maps and institutional affiliations.

Copyright: © 2021 by the authors. Submitted for possible open access publication under the terms and conditions of the Creative Commons Attribution (CC BY) license (https://creativecommons.org/licenses/by/4.0/).

1. Supplementary Materials:

Supplementary Table S1. Ultrasonography severity score.

Paramethers	Criteria	Scor
Parenchymal echotexture	Homogeneous, normal echotexture	0
	Heterogeneous echotexture	1
	Coarse echotexture	2
Nodule	None	0
	Micro nodule	1
	Macro nodule	2
	Micro and macro nodule	3

Surface or margin	Smooth	0
	Irregular	1

Supplementary Table S2. Histological liver assessment score.

**Paramethers**		**Criteria**	**Scor**
**Steatosis**	% from all tissue	≤5%	0
		5-33%	1
		33- 66%	2
		≥ 66%	3
**Fibrosis**	None		0
	Perisinusoidal or periportal		1
	Perisinusoidal and portal/periportal		2
	Bridging fibrosis		3
**Inflammation**	Lobular inflammation	None	0
		<2 foci	1
		2-4 foci	2
		>4 foci	3
	Microgranulomas	Present	1
		Absent	0
	Large lipogranulomas	Present	1
		Absent	0
	Portal inflammation	Absent	0
		None to minimal	1
		Greater than minimal	2
**Ballooning**		Absent	0
		Few balloon cells	1
		Many cells/prominent ballooning	2

Supplementary Figure S1

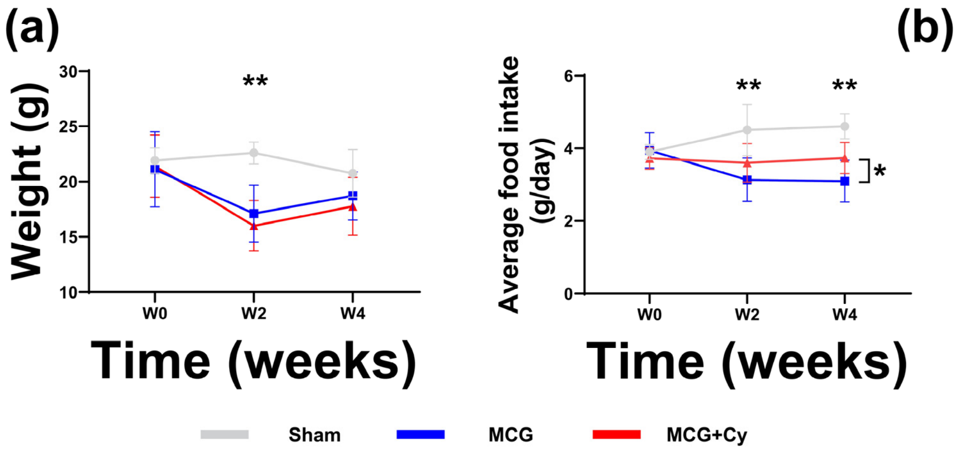

Supplementary Figure S1. Weight and food intake of the animals during the experimental period. (a) Compared to Sham, animals feed MCD food, lost approximately 20% of body mass, in the first two weeks (22.6±0.99 g, compared to 17.11±2.57 and 15.99±2.27 respectively)($p<0.01$). (b) While the Sham animals consumed around 4g a chowder per day, the MCG decreased its food intake constantly. After 2 weeks of Cereblolysin treatment, the food intake of the treated group was higher that control (3.73±0.42 vs 3.09±0.57 g/day, $p=0.011$).

Supplementary Table S3. Composition and Ingredient Amount used in the Methionine/Choline Deficient Diet

Ingredients	Weight (mg)
Sucrose	4553
Corn Starch	2000
Corn Oil	1000
Alphacel Non-Nutritive Bulk	300
AIN 76 Mineral Mix	350
Dicalcium Phosphate	30
L-Alanine	35
L-Arginine Hydrochloride	121
L-Asparagine Monohydrate	60
L-Aspartic Acid	35
L-Cystine	35
L-Glutamic Acid	400
Glycine	233
L-Histidine Hydrochloride	45
L-Isoleucine	82
L-Leucine	111
L-Lysine Hydrochloride	180
L-Phenylalanine	75
L-Proline	35
L-Serine	35
L-Threonine	82
L-Tryptophan	18
L-Tyrosine	50

L-Valine	82
DL-alpha-Tocopherol Acetate (250 u/gm)	4.84
Vitamin A Palmitate (250,000 u/gm)	0.792
Vitamin D3 (400,000 u/gm)	0.055
Ethoxyquin	0.2
Amino Acid Vitamin premix	50
